# Vertical transmission of chikungunya virus: A systematic review

**DOI:** 10.1371/journal.pone.0249166

**Published:** 2021-04-23

**Authors:** Fátima Cristiane Pinho de Almeida Di Maio Ferreira, Anamaria Szrajbman Vaz da Silva, Judith Recht, Lusiele Guaraldo, Maria Elisabeth Lopes Moreira, André Machado de Siqueira, Patrick Gerardin, Patrícia Brasil

**Affiliations:** 1 Departament of Neonatology, Universidade Federal do Estado do Rio de Janeiro, Rio de Janeiro, Brazil; 2 Independent Consultant, Maryland, United States of America; 3 Acute Febrille Illnesses Laboratory, Instituto Nacional de Infectologia Evandro Chagas, Fiocruz, Rio de Janeiro, Brazil; 4 Departament of Neonatology, Instituto Fernandes Figueira, Fiocruz, Rio de Janeiro, Brazil; 5 Groupe Hospitalier Sud Réunion, Reunion Island, France; Kobe University Graduate School of Medicine School of Medicine, JAPAN

## Abstract

**Objectives:**

To describe and estimate the frequency of pregnancy outcomes, clinical and laboratory characteristics of vertical transmission of CHIKV in the neonate.

**Study design:**

We performed a systematic review evaluating the clinical presentation of perinatally-acquired CHIKV infection in neonates. The search was performed using Medline (via PubMed), LILACS, Web of Science, Scielo, Google Scholar and Open grey to identify studies assessing vertical transmission of CHIKV up to November 3, 2020. There were no search restrictions regarding the study type, the publication date or language. Studies with no documented evidence of CHIKV infection in neonates (negative RT-PCR or absence of IgM) were excluded.

**Results:**

From the 227 studies initially identified, 42 were selected as follows: 28 case reports, 7 case series, 2 cross-sectional studies and 5 cohort studies, for a total of 266 CHIKV infected neonates confirmed by serological and/or molecular tests. The vertical transmission rate was 50% in the Reunion Island outbreak, which was the subject of the majority of the studies; the premature delivery were reported in 19 (45.2%) studies; the rate of fetal distress was 19.6% of infected babies and fetal loss occurred in 2% of the cases. Approximately 68.7% of newborns were diagnosed with encephalopathy or encephalitis after perinatally acquired CHIKV. Most of the infected neonates were born healthy, developing CHIKV sepsis clinical syndrome within the first week of life.

**Conclusions:**

We alert neonatologists to the late manifestations of neonatal CHIKV infection, relevant to the management and reduction of morbidity. A limitation of our review was that it was not possible to carry out meta-analysis due to differences in study design and the small number of participants.

## 1- Introduction

Chikungunya virus (CHIKV) is an arthropod-borne virus (arbovirus) of the alphavirus genus (*Togaviridae family*) that is believed to have originated in Africa, where it was known to circulate in sylvatic and urban cycles involving female mosquito vectors of the genus *Aedes* and non-human or human primates [1,2]. Since 2006, four lineages have been recognized: the West African, the East/Central/South African (ECSA), the Asian genotypes plus the Indian ocean lineage (IOL) that has diverged from the ECSA [3–5].

Vertical transmission was first observed in June 2005 in the CHIKV epidemic that affected more than a third of the population in Reunion Island from March 2005 to July 2006 [6,7]. Intrapartum transmission without placental infection, a direct consequence of maternal viremia and fetal or neonatal susceptibility to a given arbovirus, has been well documented for CHIKV in mouse models [8]. However, evidence of transplacental infection by the recovery of the genome of CHIKV in the amniotic fluid, the placenta and the fetal brain of stillborn fetuses in Reunion Island, when fetal loss occurred before 16 weeks of gestational age was reported. The mother’s chikungunya diagnosis was confirmed by RT-PCR detection of the viral genome in maternal blood two weeks before diagnosis of fetal loss; viral genome in the placenta and amniotic fluid confirmed transplacental transmission of CHIKV and its persistence after fetal death. The effect of the virus on the fetus was confirmed by further observations and intracellular detection of the virus in fetal tissues including brain and liver [9]. Humans transplacental CHIKV transmission though seems likely, but its pathophysiology remains unknown [6,10].

Reunion Island studies have shown that CHIKV could be transmitted vertically in about 50% of cases when the pregnant woman has high viral load during the early phase of labor. Fetal heart rate abnormalities and meconium-stained amniotic fluid were common during labor of pregnant women with CHIKV viremia. Neither postponed vaginal delivery nor cesarean section prevented fetal infection [6].

Neonatal infection can be severe and encephalopathy or encephalitis occurs in more than half cases who require intensive care [11]. The central nervous system involvement was associated with a massive brain swelling as evidenced by MRI findings [11].

The presentation, similar to bacterial sepsis poses a diagnostic challenge when maternal infection has not been diagnosed. Other differential diagnoses are those caused by other arboviruses and congenital infections of the TORCHZ group (toxoplasmosis, enterovirus, *Listeria monocytogenes*, *Mycobacterium tuberculosis*, parvovirus B19, *Treponema pallidum*, *Trypanosoma cruzi*, human immunodeficiency virus, varicella virus, rubella, cytomegalovirus, herpes simplex virus) [12].

As it is the case in pregnant women, there is no specific treatment for chikungunya in the neonate. The approach is symptomatic, focused on hydration, fever control and pain relief [13]. Vector control and personal protection are currently the only chikungunya prevention measures available [13].

The limited knowledge about the clinical manifestations in the neonate and pregnancy outcomes of CHIKV vertical transmission prompted us to conduct this study. We performed a systematic review of the available literature reporting the clinical and laboratory characteristics of CHIKV vertical transmission in the neonate aiming at gathering the best evidence to critically evaluate the approach following CHIKV infection in pregnancy.

## 2- Materials and methods

The review was conducted according to a pre-established protocol and described following PRISMA (Preferred Reporting Items for Systematics Reviews and Meta-Analyzes) recommendations [14].

### 2.1 Search

The literature search was performed using Medline (via PubMed), LILACS, Web of Science, Scielo, Google Scholar and Open grey to identify studies assessing vertical transmission of chikungunya virus up to November 3, 2020. Additional articles not found by the above searches were identified from the reference lists of the selected articles. No study, according to the inclusion criteria, was identified using the open gray search tool.

The search descriptors used for Medline were as follows: (((congenital chikungunya) AND (((newborn) OR (((infant OR neonatal))) AND (((mother-to-child OR congenital OR vertical transmission)))) AND chikungunya). The complete search strategies were adapted according to each data base.

### 2.2 Selection

Articles were selected for inclusion by two independent authors (FF and AS), with differences resolved after discussion and consensus with a third author (PB). We included studies of different design that evaluated perinatal transmission of CHIKV regarding its clinical presentation and therapeutic approach, with confirmed laboratory diagnosis in neonates (RT-PCR and/or IgM serology positive for CHIKV) and with clinical, epidemiological and/or confirmed laboratory diagnosis in pregnant women. There were no restrictions regarding publication date or language.

Initially titles and abstracts were read to exclude those that did not fall into the eligibility criteria. Subsequently, the full texts potentially eligible for inclusion were read and the same inclusion and exclusion criteria were applied. In addition, we excluded studies with no documented evidence of CHIKV infection in neonates (negative RT-PCR or absence of IgM).

### 2.3 Data extraction and quality assessment

The data were extracted by two authors (FF and AS), disagreements were resolved by consensus reached through a third author (PB). A standardized form developed for this review and pre-tested on three articles was used for extracting the data. The form contained the following sections: study identification (study title, author, journal, year of publication, country, language and financial institution); characteristics of the study (design, number of patients and study period); characteristics of the study population (**pregnant women:** diagnostic methods, clinical parameters, laboratory and treatment; **neonates:** age, gender, gestational age, birthweight, delivery type, place of hospitalization, diagnostic methods, clinical parameters, laboratory and treatment).

We included studies with clinical and laboratory neonatal data, as well as age in days at onset of illness and outcome in the neonate such as sequelae or death. From the selected articles, clinical, laboratory and perinatal variables of CHIKV infection were collected for pregnant women and neonates.To describe chikungunya evolution during pregnancy, the following variables were extracted: gestational age at onset of maternal disease, time of onset of disease in relation to delivery, type of delivery and outcome of pregnancy, such as death of the pregnant woman, premature delivery/preterm birth, fetal distress, miscarriage, or stillbirth.

Assessment of the methodological quality of the included studies was based on the Methodological Index for Non-randomized Studies (MINORS). The instrument consists of 12 items, the first eight being specific for non-comparative studies [15].

Because of differences in studies design and the small number of participants, a meta-analysis was deemed inappropriate and we focused on describing the studies in evidence with their results and a qualitative synthesis.

## 3- Results

**Fig 1:**
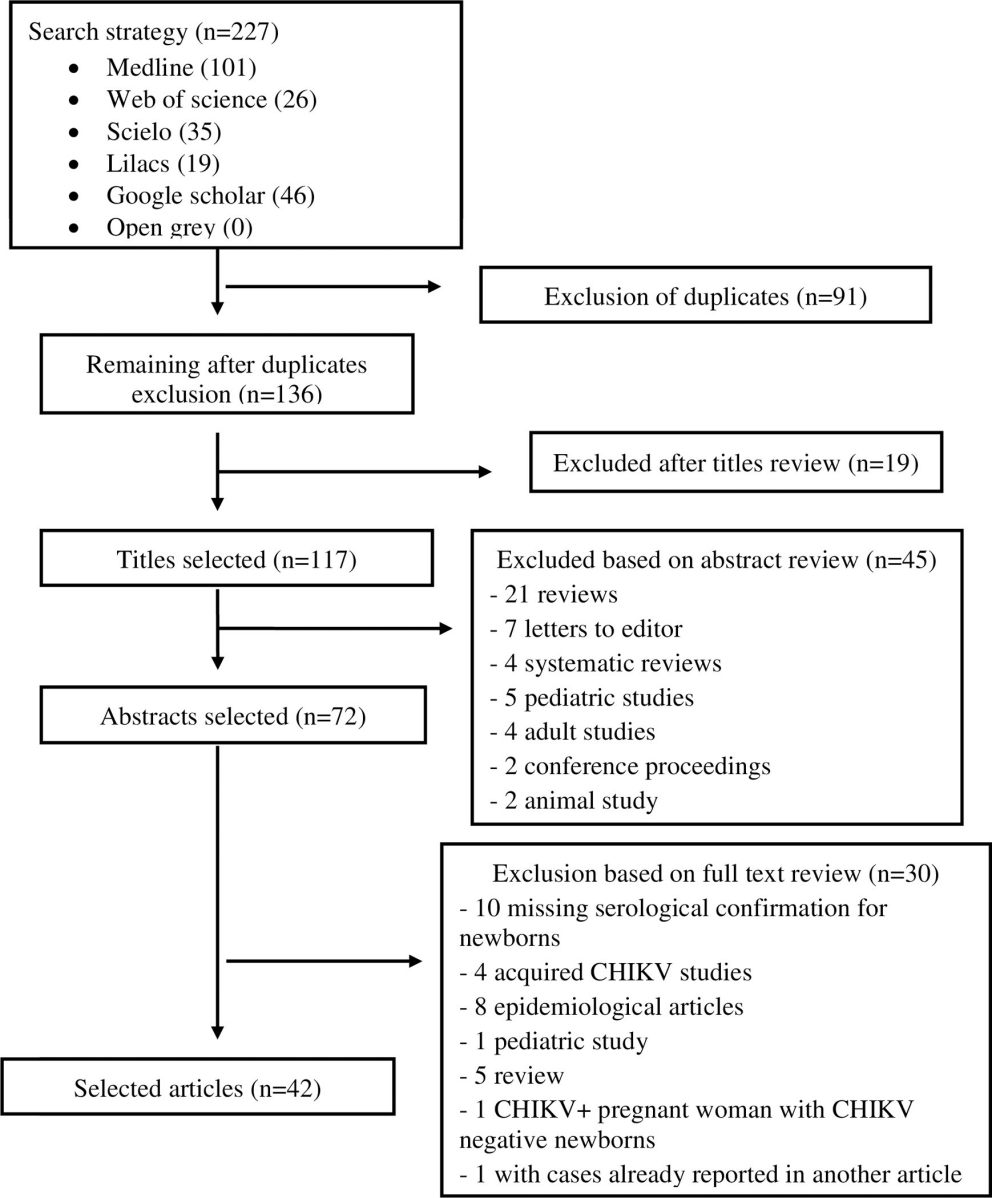
Flowchart of review process.

The literature search retrieved 227 publications; after exclusion of 71 duplicates, 136 titles were evaluated regarding the established inclusion and exclusion criteria. Of these, 64 were excluded based on the title (19) and abstract (45) (Fig 1). The final selection resulted in 42 articles: 28 case reports, seven case series, two cross-sectional study and five cohort studies. No new articles were added after searching the reference section of the selected articles (Fig 1).

**Fig 2:**
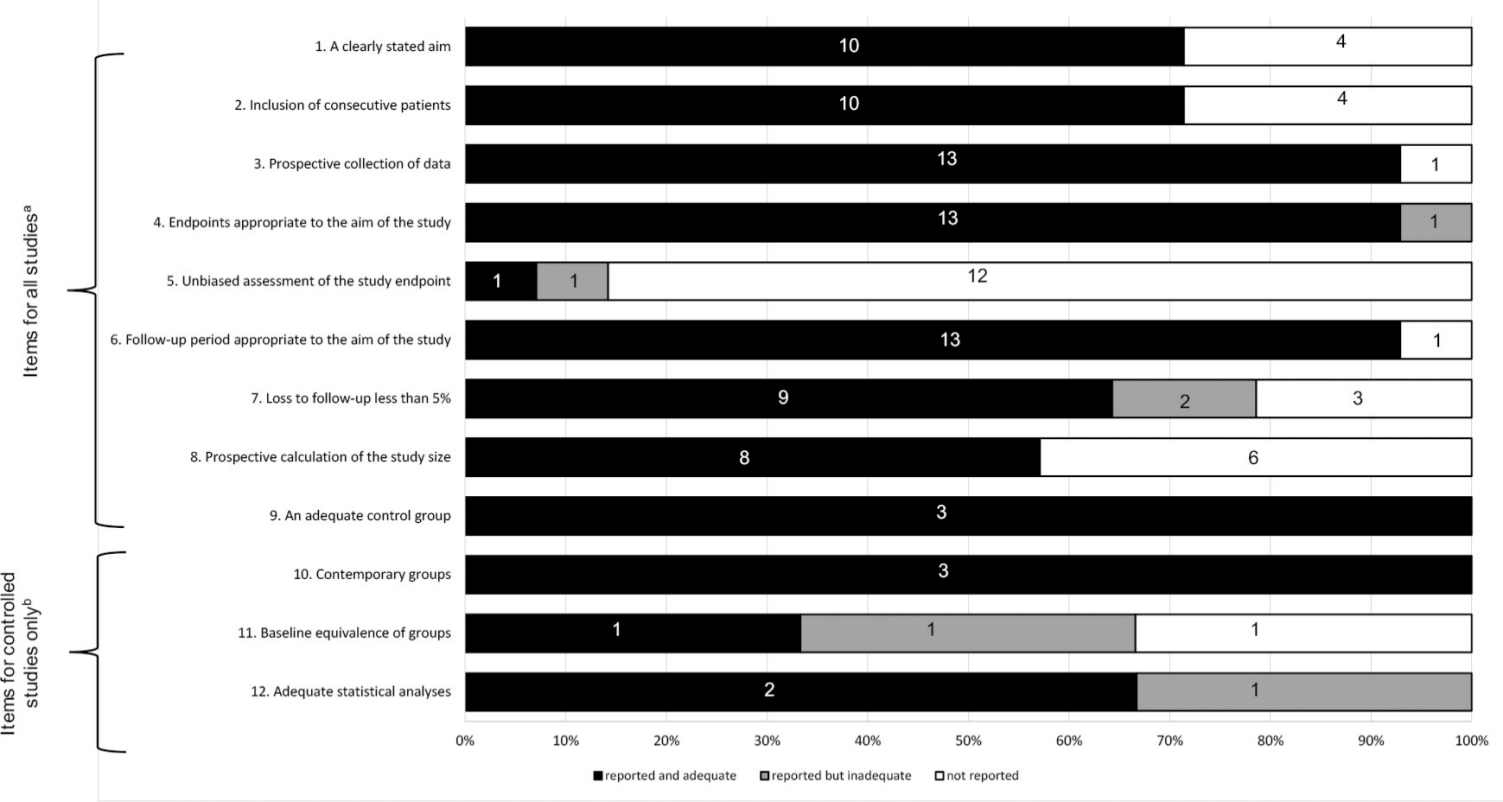
Quality assessment of the studies included in the systematic review based on Methodological Index for Nonrandomized Studies (Slim et al, 2003) [15]. (A) Quality items for all observational studies. (B) Quality items for comparative studies.

The quality of articles was assessed by MINORS instrument for all observational studies except for case reports. More than 90% of the studies presented endpoints to their aim and follow-up period appropriate and all reported an adequate control group. More than half of studies report the inclusion of consecutive patients. About 90% of the studies did not report an unbiased assessment of the study end point. Comparative studies (n = 3) achieved good quality criteria, more than 60% presented adequate statistical analyses (Fig 2).

In this systematic review of 42 studies that included 266 fetuses or neonates infected with CHIKV by vertical transmission, neonatal infection was severe, manifesting with neurological disease hospitalized in intensive care in 76.5% of cases. The development of life-threatening neonatal symptoms was not associated with the severity of maternal symptoms. From the maternal inoculum, it takes three to seven days for the CHIKV load to reach a significant level able to cause clinical disease. Whether the vertical transmission occurs through the syncytiotrophoblast or *per mucosa* during childbirth remains undiscovered. No infection was demonstrated through breastfeeding, which was postponed until the clearance of maternal symptoms.

### 3.1 Characteristics of selected studies

The selected studies were published between 2006 and 2020 and conducted between 2005 and 2019 (Table 1). All studies included newborn infants hospitalized in neonatal intensive care units (NICUs), with clinical chikungunya and laboratory evidence of viral infection. Cases without laboratory confirmation or postnatal chikungunya were excluded from the analysis.

**Table 1:** Characteristics of included studies.

Study [reference], Author, Year	Study period, location,	Sample size (N)	Type of study	Pregnant woman CHIKV+ diagnosis	Number of vertical transmission cases, diagnosis*
[16], Correa, 2020	ND, Brazil	6	Case report	Clinical-laboratory	6, IgM+, RT-PCR
[17], Jebain, 2020	ND, Brazil	1	Case report	Clinical-laboratory	1, IgM+, RT-PCR
[18], Correa, 2020	ND, Brazil	3	Case report	Clinical-laboratory	3, IgM+, RT-PCR
[19], Shen, 2020	2019, China	3	Case report	Clinical-laboratory	3, IgM+, RT-PCR
[20], Chandramathi, 2020	ND, India	1	Case report	Clinical epidemiological	1, IgM+
[21], Di Maio Ferreira, 2019	2016, Brazil	1	Case report	Clinical-laboratory	1, IgM+, RT-PCR
[22], Dorléans, 2018	2013–2015; Martinique and Guadaloupe	15	Cross-sectional	Clinical-laboratory	15,IgM+, RT-PCR
[23], Kumar, 2018	2006, India	2	Case report	Clinical-laboratory	2, IgM+
[24], Kumar, 2018	2016; India	6	Case report	Clinical-laboratory	6, IgM +, RT-PCR
[25], Oliveira, 2018	2017; Brazil	1	Case report	Clinical-epidemiological	1, IgM +
[26], Van Enter, 2018	2018; Island of Curaçao	14	Case report	Clinical-laboratory	3, IgM+
[27], Leitão, 2018	2016; Brazil	5	Case report	clinical-laboratory	5, RT-PCR
[28],Maria A, 2018	Aug–Nov 2016; India	10	Case series	clinical-laboratory	10, IgM +
[29], Cardona- Correa, 2017	ND; Colombia	1	Case report	clinical-epidemiological	1, IgM+
[30], Evans-Gilbert, 2017	2014; Jamaica	2	Case report	clinical-laboratory	1, RT-PCR
[31], Ramos, 2018	Feb 2016; Brazil	1	Case report	clinical-laboratory	1, RT-PCR
[32], Alvarado-Socarras, 2016	Aug 2014-Feb 2016; Colombia	2	Case report	clinical-epidemiological	1, IgM+
[33], Torres, 2016	2014–2015; Brazil, El Salvador, Dominican Republic, Venezuela	169	Case Series	clinical-laboratory	55, RT-PCR
[34], Karthiga, 2016	ND; India	2	Case report	clinical-laboratory	2, IgM+
[35], Nigam, 2016	ND; India	1	Case report	clinical-laboratory	1, RT-PCR
[36],Lyra, 2016	2014–2016; Brazil	2	Case report	clinical-laboratory	2, RT-PCR
[37], Bandeira, 2016	2015; Brazil	1	Case report	clinical-epidemiological	1, RT-PCR
[38], Pinzon-Redondo, 2016	Sept-Dec 2014; Colombia	54	Case series	ND	2, RT-PCR
[39], Vasani, 2016	ND; India	2	Case report	clinical-laboratory	1, IgM+
[40], Muñoz, 2014	2014; Colombia	18	Case report	ND	2, RT-PCR
[41], Villamil-Gomez, 2015	Sept 2014-Feb 2015; Colombia	8	Case series	clinical-laboratory	8, IgM+ and RT-PCR
[42], Taksande, 2015	ND; India	1	Case report	clinical-epidemiological	1, IgM+
[43], Gérardin, 2014	2005–2006; Reunion Island	33	Cohort	clinical-laboratory	33, IgM+ and RT-PCR
[44], Ramful, 2014	2005–2006; Reunion Island	653	Cohort	clinical-laboratory	1, IgM+ and RT-PCR
[45], Shenoy, 2012	ND; India	2	Case report	clinical-laboratory	2, RT-PCR
[46], Boumahni, 2012	June 2005-March 2006; Reunion Island	20	Case report	clinical-laboratory	1, IgM+ and RT-PCR
[47], Shrivastava, 2011	ND; India	1	Case report	clinical-laboratory	1, RT-PCR
[48], Boumahni, 2011	ND; Reunion Island	1	Case report	clinical-epidemiological	1, IgM+ and RT-PCR
[49], Sharanabasavesh, 2011	2006–2010; India	8	Case series	Clinical-laboratory	8, IgM+ and RT-PCR
[50], Fritel, 2010	April-Nov 2006; Reunion Island	914	Cohort	clinical-laboratory	1 newborn, 2 stillborn, IgM+ and RT-PCR
[51], Senanayake, 2009	April-Oct 2007; Sri Lanka	50	Case series	clinical-laboratory	7, IgM+
[52], Rao, 2008	2006; India	2	Case report	clinical-epidemiological	2, RT-PCR
[6], Gérardin, 2008	June 2005-Dec 2006; Reunion Island	739	Cohort	clinical-laboratory	19, IgM+ and RT-PCR
[53], Ramful, 2007	March 2005-April 2006; Reunion Island	38	Case series	clinical-laboratory	38, IgM+ and RT-PCR
[9], Touret, 2006	Dec 2005-Feb 2006; Reunion Island	3	Case report	clinical-laboratory	3 fetuses <22 weeks, RT-PCR
[54], Paquet, 2006	March 2005-Jan 2006; Reunion Island	6	Cross-sectional	clinical-laboratory	6, IgM+ and RT-PCR
[10], Lenglet, 2006	June 2005-Feb 2006; Reunion Island	160	Cohort	clinical-laboratory	16, IgM+ and RT-PCR

ND: No data.

* Diagnosis was by serology (IgM+) and/or RT-PCR.

Most studies (n = 35; 83,3%) were case reports and case series [9,16–21,23–42,45–49,51–53]. The clinical and perinatal manifestations of pregnancy and the outcome of gestation were described in ten studies [9,10,16–21,23,25,27,49–51]. Stillbirths and miscarriage associated with CHIKV vertical transmission with laboratory evidence were reported in three studies [9,50,54].

The 42 selected studies included 266 cases of vertical transmission and were conducted in countries that suffered from chikungunya epidemics, mostly Central and South America and the Caribbean, India and Reunion Island (French overseas department). Most studies in Central and South America and the Caribbean were case reports. A case series study was conducted in Latin America, which covered cases in four maternity hospitals from three different countries: Colombia, El Salvador and the Dominican Republic. Ninety-nine cases from the Dominican Republic and 35 cases from Colombia were excluded from the systematic review because they lacked laboratory confirmation [33]. Vertical transmission rates ranged from 27.7% to 48.7% in two selected studies [6,33].

### 3.2 Pregnancy outcome, clinical and perinatal characteristics of the pregnant women (Table 2)

All included studies (n = 42) reported CHIKV vertical transmission confirmed by laboratory and clinical-epidemiological evidence in pregnant women (Table 1). The maternal infection diagnosis was confirmed by molecular and serological tests in 31 (73.8%) studies [6,9,10,16–28,30,31,33–35,39,41,43–45,47–51,53,54] and in 243 (96.8%) women.

**Table 2:** Characteristics of pregnant women, neonates and their outcomes.

Study [reference], Author, Year	Pregnant women (N), Delivery type, Gestational age (weeks), Chikungunya start (days before delivery)	Pregnant woman manifestations (clinical and laboratory)	Fetal distress, gestation outcome	Neonates (N), Chikungunya start (days after birth), neonatal deaths	Neonatal manifestations (clinical and laboratory); sequelae
[16], Correa, 2020	6, C (4), V (2), 37 sem (3), 34 sem 5 d, 35 sem and 36 sem, 1 to 7	Fever, arthralgia, rash	ND, PMT	6, 4 to 10, N	Fever, hypoactivity, seizure, sepsis, irritability, thrombocytopenia, ND
[17], Jebain, 2020	1, ND, Term, after birth	Fever, arthralgia	ND	1, 7, N	Bullous dermatitis, ND
[18], Correa, 2020	3, C (2) and V (1), 36 (1) and 37 (2), at birth	Fever, arthralgia, rash	ND, PMT	3, 4,5 and 6, N	Hypoactivity, fever, rash, suctioning difficult, thrombocytopenia, longitudinal brain magnetic resonance with brain impairement; ND
[19], Shen, 2020	2, C, PMT and Term, at birth	Fever, arthralgia, rash, myalgia	ND, PMT	3, 5 and 3, N	Fever, rash, respiratory failure, jaundice, arthralgia and hypoactivity; ND
[20], Chandramathi, 2020	1, ND, ND, 7	Fever	ND	1, 15, N	Apnea,fever,seizure,sepsis, jaundice, respiratory failure, thrombocytopenia, ND
[21], Di Maio Ferreira, 2019	1, C, Term, at birth	Fever, arthralgia, rash	Y, ND	1, 6, N	Rash, fever, arthralgia, arthritis, hypoactivity, hyperpigmentation, bullous dermatitis; none
[22], Dorléans, 2018	15, ND, ND, 6	ND	ND	15,ND; N	ND
[23], Kumar, 2018	2,V, ND, 4 and 1	Fever, arthralgia, rash	ND, PMT (1)	2, 5 and 4, N	Rash, fever, arthralgia, arthritis, hypoactivity, hyperpigmentation, hemodynamic instability, respiratory distress, seizure; ND
[24], Kumar, 2018	6, ND, ND, ND	ND	ND	6, ND, N	ND
[25], Oliveira, 2018	1, C, 34, 10	Itching, arthralgia, arthritis, fever, headache, seizure, abdominal pain, diarrhea, cough, dyspnea and arterial hypertension, respiratory failure; thrombocytopenia and neutrophilia	ND, PMT and death	1, at birth, 1	Apnea,fever,seizure,sepsis, jaundice, gastrointestinal bleeding, pulmonary hemorrhage, respiratory failure; death
[26], Van Enter, 2018	14, C (2) and V(1), 35 sem 2 d (1) and ND (13), 1 (2) and 7 (11)	ND	Y, PMT (1)	3, at birth (1) 4 (1), 5 (1); 1	Intracranial hemorrhage, fever, rash, seizure, irritability, suctioning difficult; death
[27], Leitão, 2018	5, ND, ND, ND	Rash,arthralgia, fever,bullous dermatitis	ND	5, 2 to 4, N	Fever,hypoactivity,encephalitis, meningoencephalitis, seizure, irritability, bullous dermatitis, hyperpigmentation intracranial hemorrhage, arthralgia thrombocytopenia; ND
[28],Maria A, 2018	10, ND, ND, ND	ND	ND	10,ND,N	Fever, hypoactivity, respiratory distress, suctioning difficult, hemodynamic instability, encephalitis, seizure, hyperpigmentation, jaundice, thrombocytopenia, leukopenia; Neurological
[29], Cardona- Correa, 2017	1, C, 35, 3	Rash, headache, arthralgia	N, PMT	1, 4, N	Rash, irritability, hypoactivity, respiratory failure, intracranial hemorrhage, thrombocytopenia; neurological (optic nerve atrophy, microcephaly, epilepsy and cerebral palsy)
[30], Evans-Gilbert, 2017	1, V, 39, ND	Fever, arthralgia	N, N	1, 5, N	Rash, fever, thrombocytopenia, elevated GOT; none
[31], Ramos, 2018	1, C, 35,3	Fever, disseminated intravascular coagulation, arthralgia, abdominal pain, renal failure, rhabdomyolysis	Y, PMT	1, 3, 1	Fever, hypoactivity, apnea, respiratory failure, cyanosis, hemodynamic instability, elevated GOT; none
[32], Alvarado-Socarras, 2016	1, C, 38,1	Rash, fever, pelvic pain, edema	N, N	1, 3, N	Rash, fever and irritability, thrombocytopenia, elevated GOT; none
[34], Karthiga, 2016	2, ND ND, ND	ND	ND, ND	2, 5 and 8, N	Rash, fever and irritability; ND
[33], Torres, 2016	55, ND ND, ND	ND	ND, PMT	55, 5 (3–9), N	El Salvador and Colombia: fever, hypoactivity, suctioning difficulty, hemodynamic instability, respiratory insufficiency, meningoencephalitis, exanthema, bullous dermatitis, hyperalgesia, limb edema, myocarditis thrombocytopenia, anemia, pleocytosis; ND
[35], Nigam, 2016	1, V 36, ND	Fever	ND, PMT	2, 5, N	Facial hyperpigmentation, respiratory insufficiency, convulsion, intracranial hemorrhage, thrombocytopenia, anemia; neurological
[36],Lyra, 2016	1, C 36, 15	Fever and arthralgia, thrombocytopenia	Y, PMT	1, birth day, N	Tachypnea and pericardial effusion; none
[37], Bandeira, 2016	2, V and ND 38 and 41, 2	One case rash, fever and arthralgia, another ND	N, N	2, 1–4, N	One case rash, fever, respiratory failure, hemodynamic instability, hepatosplenomegaly and another fever, hypoactivity, hemodynamic inatability and grade I intracranial hemorrhage; none
[38], Pinzon-Redondo, 2016	1, V ND, 4	Rash and fever	N, ND	1, 4, N	rash, fever, hypoactivity, convulsion, encephalitis; ND
[39], Vasani, 2016	ND, ND ND, ND	ND	ND, ND	2, 5, N	rash, fever and diarrhea, thrombocytopenia, lymphopenia; ND
[40], Muñoz, 2014	1, V ND, 7	Fever	ND, ND	1, birth day, N	Respiratory insufficiency and hyperpigmentation; ND
[41], Villamil-Gomez, 2015	1, V 38, 3	Fever, headache and myalgia	N, N	1, 3, N	Fever and irritability; ND
[42], Taksande, 2015	1, ND ND, At birth	ND	ND, ND	1, 6, N	ND; ND
[43], Gérardin, 2014	ND, ND ND, ND	ND	ND, PMT	33, ND, N	ND; neurological
[44], Ramful, 2014	7, V (2), C (5) 32 to 39, At birth	Rash, fever and arthralgia, edema and headache	N, PMT (3)	8 (one twins), ND, 3	Exanthema, fever, bullous dermatitis, respiratory failure, meningoencephalitis, gastrointestinal bleeding, hyperalgesia, edema, myocarditis, pericarditis, hemodynamic instability and enterocolitis, thrombocytopenia; none
[45], Shenoy, 2012	1, C 36, At birth	Fever	Y, PMT	1, 4, N	Respiratory insufficiency, hemodynamic instability and septic shock; neurological
[46], Boumahni, 2012	2, C ND, ND	Fever and arthralgia	N, N	2, 5, N	Both cases hyperpigmentation, convulsion and apnea, only case 2 respiratory insufficiency; neurological (case 2 optic nerve atrophy)
[47], Shrivastava, 2011	1, C ND, ND	Thrombocytopenia	N, N	1, 5, N	Fever, seizure, hyperalgesia, apnea, respiratory failure, thrombocytopenia; neurological
[48], Boumahni, 2011	1, C 35, 3	Fever and arthralgia	Y, PMT	1, birth day, N	Fever, rash, hyperpigmentation, sucking difficulty, apnea, thrombocytopenia and elevated GOT; none
[49], Sharanabasavesh, 2010	8, ND, 1premature and 7 term, 1	Fever	ND	8, 5, N	Sucking difficulty, seizure, fever, encephalitis, hyperpigmentation, hepatomegaly, respiratory failure, thrombocytopenia, ND
[50], Fritel, 2010	3, ND ND, 2 (one newborn), 25 and 70 (two stillborns)	ND	ND, 2 stillborns	1, 3, N	ND; none
[51], Senanayake, 2009	7, ND ND, 0 (4 cases) and 1st, 2nd and 3rd trimestres	Fever and arthralgia ine case, other cases ND	ND, ND	7, 2 (one case) ND others, N	One case fever, hyperpigmentation, irritability, hypoactivity and myocarditis, 3 cases hyperpigmentation 3 cases ND; ND
[52], Rao, 2008	19, V (9), C (10) 35–40, At birth	ND	Y (14) ND (5), ND and PMT	19, 4 (3–7), N	Intracranial hemorrhage, disseminated intravascular coagulation, hyperalgesia, hematemesis, hypovolemic shock, respiratory insufficiency, thrombocytopenia, lymphopenia, anemia, elevated GOT, hypocalcemia, altered coagulogram, fever, rash, convulsion, hypoactivity; suction difficulty; neurological and blindness
[6], Gérardin, 2008	38, V (12), C (26) 35–41, 0 (18) and 4 until 2 days post-partum, 2 asymptomatic	ND	Y (17) ND (21), ND and one PMT	38, 4 (3–7), 1	Fever, exanthema, hyperpigmentation, hyperalgesia, gastrointestinal and conjunctival hemorrhage, respiratory failure, necrotizing enterocolitis, diarrhea, suctioning difficulty and hemodynamic instability, thrombocytopenia, lymphopenia, hypokalemia, altered coagulogram; neurological
[53], Ramful, 2007	2, V and C ND, 1 and 4	Fever, arthralgia and headache	ND, N	2, 3 and 5, N	One case fever, rash, irritability, sucking difficulty, apnea, another fever, hyperpigmentation, mucositis, difficulty sucking, inadequate weight gain; none
[9], Touret, 2006	3, ND ND, ND	ND	ND, miscarriage	NA	NA
[54], Paquet, 2006	3, V 12 to 15, at 12 (1 case) and 15 (2 cases) weeks gestation	Rash, fever, arthralgia and headache	ND, miscarriage (3)	N, N, N	None; none
[10], Lenglet, 2006	16, V (9), C (7) ND, At birth	Rash, fever, arthralgia	Y (10) ND (6), N	16, 3–7, N	Fever, ND

ND: No data; NA: Not applicable; PMT: Premature; FD: Fetal distress; GOT: Glutamic oxaloacetic transaminase; C: Ceasarean section, V: Vaginal delivery; Y: Yes; N: None.

The CHIMERE cohort study on Reunion Island reported five miscarriages and five stillbirths; of the later tested by RT-PCR performed on placenta and amniotic fluid (AF) samples, two were CHIKV positive, one was negative and the remaining two were not tested [50]. One miscarriage (<22 weeks) was tested negative. For the two fetuses tested positive in the amniotic fluid, maternal symptoms of CHIKV infection began about 25 to 70 days before fetal loss. A previous report from Reunion Island identified three fetal losses from pregnant women who presented CHIKV clinical manifestations at about two, three and four weeks respectively before the detection of fetal death on routine ultrasound examination. CHIKV infection was confirmed by molecular examination (RT-PCR) of the placenta, AF, and fetal brain [9]. A case series study during an epidemic in Sri Lanka reported 50 pregnant women with serological proof of CHIKV infection, and among these one single case of first trimester miscarriage and one stillbirth at 33 weeks [51]. The fetal losses were not virologically analyzed in this study.

#### 3.2.1 Pregnancy outcomes (Table 2)

In two studies, a case of maternal death occurred. In one them, the death occured four days postpartum, with rhabdomyolysis, disseminated intravascular coagulation (DIC) and acute renal failure [30]. In the other one, a 28-year-old pregnant woman with hypertension presented with symptoms compatible with an arboviral disease at 34 weeks’ gestation. She developed preeclampsia with severe respiratory failure which resulted in the emergency cesarean section, and the patient died 12 days after the onset of symptoms [25].

Two cohort studies [6,50] reported six miscarriages (<22 weeks of gestational age) and two stillbirths (> 22 weeks of gestational age) [50]. Premature deliveries/preterm births were reported in 19 (45.2%) studies. One of these showed a median gestational age at childbirth of 38 weeks (35–41 weeks) [6]. Latin American studies reported a mean gestational age of 38 and 39 weeks [33,41]. Among these studies, there were related 18 premature deliveries/preterm births and 58 full-term deliveries/full-term neonates. No congenital malformation was described.

#### 3.2.2 Perinatal characteristics (Table 2)

In one single case report, the onset of chikungunya illness for the pregnant woman began 15 days before delivery [35]. In twelve studies (28.5%), the timing of onset of symptoms before childbirth was not reported. The other studies showed that symptoms started during the perinatal period (from 10 day before delivery until 2 day postpartum), therefore maternal chikungunya onset ranged from 15 days before delivery to two days after delivery with 158 mothers of infants infected with CHIKV presenting chikungunya signs during the perinatal period. The gestational age of chikungunya infection in the pregnant woman was not described in 20 (47.6%) of the studies. Among others, the majority of CHIKV infection was reported in the second and third trimester, mainly in the intrapartum period [50,51,54]. In 19 studies (45,2%) childbirth by cesarean delivery was postponed, whereas in 16 studies (38%) it was vaginal delivery. Fetal distress was observed in 9 (21.4%) studies, of which 66,4% were deliveries by cesarean section. The rate of fetal distress among infected pregnant women was 49 (19.6%). In total, 75 cases of cesarean delivery were reported, 48 of which were due to fetal distress. The number of vaginal deliveries was 96 cases and in 158 cases the birth route was not reported.

### 3.3 Clinical and perinatal characteristics of the neonates (Table 2)

All studies described newborn infants with CHIKV clinical manifestations and laboratory, serological and molecular tests confirming the vertical transmission. The clinical and laboratory evidence, and in some cases the outcome of vertical transmission and the therapeutic approach for these neonates were reported. A total of 266 confirmed infected neonates were reported by serological and/or molecular examination, in addition to two stillbirths and four miscarriages.

#### 3.3.1 Clinical characteristics (Tables 2 and 3)

Neonatal sepsis was the most common CHIKV clinical presentation in the neonate; it was reported in 36 (85.7%) studies and 250 cases.

**Table 3:** Characteristics of 42 mother-to-child congenital chikungunya studies.

Clinical findings	Manifestation	N of studies (%)
**Fever**		24 (57.1%)
**Neurological**	Hipoactivity Irritability Poor feeding Encephalopaty/encephalithis Meningoencephalitis Seizures Intracranial hemorrhage	24 (57.1%)
**Dermatological**	Maculopapular eruption Hyperpigmentation Bullous dermatoses Skin scaling	24 (57.1%)
**Respiratory**	Apnea Respiratory failure	24 (57.1%)
**Hematological**	Thrombocytopenia Disseminated coagulation intravascular	17 (40.4%)
**Cardiovascular**	Hemodynamic instability Myocarditis Pericardial effusion Pericarditis	11 (26.1%)
**Gastrintestinal**	Enterocolitis Diarrhea Mucositis Gastrointestinal bleeding	9 (21.4%)
**Sequels**	Neurological	9 (21.4%)
**Skeletal muscle**	Hyperalgesia Diffuser lower limb edema	7 (16.6%)
**Death**		5 (11.9%)
**Miscarriages**		2 (4.75%)
**Stillbirth**		2 (4.75%)

In all studies the neonates were admitted to the NICU. The first signs and symptoms appeared between the first and the fifteenth day of life. The signs and symptoms reported in the 42 studies were in decreasing order: 24 (57.1%) presenting fever and rash, (187 cases with fever and 147 cases with rash); in 24 (57.1%) studies there were 183 cases with neurological signs characterized by irritability, seizures, meningoencephalitis, hypotonia and encephalitis, and 111 of respiratory failure characterized by apnea and the need for oxygen therapy; 11(26.1%) studies reported 137 cases of hemodynamic instability; nine (21.4%) studies reported 65 cases with gastrointestinal findings; seven (16.6%) studies reported 123 cases of hyperalgesia. Other signs and symptoms such as septic shock, bullous dermatosis, hyperpigmentation, intracranial hemorrhage, pericarditis, myocarditis, pericardial effusion, coronary dilatation, diarrhea and necrotizing enterocolitis were also reported (Table 2).

Nosocomial sepsis complicating maternal-neonatal chikungunya was reported twice with detection of *Staphylococcus sp*. and *Klebsiella pneumonia* in blood culture, and CHIKV viral genome by RT-PCR as well [36,53].

### 3.4 Laboratory characteristics (Table 2)

There were few laboratory findings reported. Seventeen studies (40.4%) reported 74 cases of thrombocytopenia (5,000–150,000 mm^3^) and five (11.9%) studies reported 41 cases of increased aspartate aminotransferase. Other relevant laboratory findings were anemia and prothrombin time enlargement.

### 3.5 Therapeutic approach

Therapeutic management of the neonates consisted of symptomatic measures in all studies, all cases being admitted to the NICU for supportive cares due to the severity of chikungunya presentation. The median length of hospital stay was 18 days (3–69 days). The use of antibiotic therapy was reported in 23 (54.7%) studies, corresponding to 28 cases. Respiratory failure and apnea, leading to invasive mechanical ventilation was reported in 15 (35.7%) studies or 48 cases. Transfusion of blood products (fresh frozen plasma and/or platelet concentrates and/or packed red blood cells) was performed in seven (16.6%) studies or 45 neonates. The use of vasoactive amines and/or volumetric expansion in patients with hemodynamic instability was reported in six (14.2%) studies or 24 cases. Pain assessment was done through a pain scale for the neonates in one study [9]. Neonates received analgesics for pain relief in three studies [5,9,40].

### 3.6 Neonate outcomes (Table 2)

Neurological sequelae (cognitive and/or motor and/or ophthalmologic dysfunction) of CHIKV vertical transmission were reported among infants in nine (21.4%) studies for a total of 106 cases; no information was reported in the remaining studies (n = 33; 78.5%). Few studies reported the infant follow-up time by a multidisciplinary team. Five studies (11.9%) described a total of 9 neonatal deaths; three of these newborns developed necrotizing enterocolitis, and the others had septic shock as the cause of death.

Only in twelve studies (28.5%), magnetic resonance imagery (MRI) was done, demonstrating the evolution of lesions from edema to cavitation or subcortical atrophy [5,16,18,29,55].

## 4- Discussion/Conclusions

This review confirms the severity of neonatal cases due to the vertical transmission of CHIKV. Maternal-neonatal chikungunya presents as a neurological disease or a neonatal sepsis that requires intensive support and upstream, the surveillance of CHIKV positive pregnant women all throughout pregnancy, and especially, during childbirth.

Vertical transmission of CHIKV was first reported during the 2005–2006 epidemic on Reunion Island, the location of most of the studies included here. It was demonstrated that the effects of CHIKV infection occurred almost invariably during maternal viremia in the peripartum period with a vertical transmission rate of about 50% and with a high viral load in the placenta [7,56]. As newborns infected in the peripartum period are born with very low or even undetectable viremia, placental microtransfusion seems unlikely as expected neonatal viremia would be close to that of the mother. In all cases, vertical transmission occurred regardless of the delivery route (75 cesareans and 96 natural deliveries). Premature delivery was related in 45.2% of studies. Most of the infected neonates were born healthy, developing CHIK clinical signs and symptoms within the first week of life. Fetal distress was reported in 25% of infected babies and was the cause of a cesarean delivery indication, reported in 69.2% of cases in one study [6]. Fetal loss (miscarriage and stillbirth) was reported in 4.75% of the studies. These rates were reported to be much lower in a recent meta-analysis that evaluated the risk for mother-to-child transmission (15.5%), antepartum fetal deaths (0.3%), and neonatal deaths (0.6%) [57]. In our review, the vertical transmission rate could not be estimated because neonatal confirmed CHIKV infection was an inclusion criteria of the study. The quantitative analysis of the study is impaired since the denominator of the number of cases on Reunion Island is unknown due to the absence of birth records from other places on the Island, with only data from the Saint Pierre region in the south of the Island. Saint Pierre is responsible for 40% of the total births on the island, but the epidemic also affected the eastern region and we do not have these data. In view of the above, the quantitative analysis data do not correspond to the reality of the epidemic in Reunion Island, impairing a meta-analysis.

The approach of this systematic review differs from the recent meta-analysis, published in 2018, as the latter is a study of an epidemiological nature while this review is aimed at the medical public and provides additional information for obstetricians and neonatologists complementing the meta-analysis information.

A limitation of this review was that the designs of the papers that we selected differed, with the majority being case reports, which hampered quantitative analysis. A strength is the clinical characterization of signs and symptoms associated with the vertical transmission of CHIKV, which is critical for improving the diagnosis of suspected cases and the appropriate therapeutic management of infected neonates. A novel contribution of this study is that it provides insights about arboviruses in endemic areas where the lack of adequate diagnosis and clinical management are associated with increased morbidity. Our review confirms that CHIKV can cause significant morbidity and also death in infected fetuses and neonates after maternal infections during gestation, which supports the previous meta-analysis [57].

To the best of our knowledge, there are no cohort studies in the literature that has reported vertical transmission with other CHIKV genotype than the IOL (mutation of the ECSA genome virus). Only three cohort studies [6,50,51] described vertical transmission. The main limitations of this review were the restriction of data to areas currently suffering or threatened by CHIKV epidemics; and the heterogeneity of mostly selected case reports and series, that prevented meta-analysis or statistical comparisons. The handling of these vertical cases is based on the opinion and experience of experts rather than higher rated evidence.

Despite laboratory confirmation of CHIKV vertical transmission in all neonates, some pregnant women only had clinical-epidemiological diagnosis, demonstrating the difficulty of simultaneous viral detection or serological conversion in the mother-child pair [33].

No correlation of the presence or severity of maternal symptoms with neonatal outcomes was reported.

The main clinical manifestations in neonates were those of neonatal sepsis: hypoactivity, irritability, sucking difficulty, apnea, hemodynamic instability and respiratory failure. In addition, most of the neonates presented fever, rash, hyperpigmentation, hyperalgesia, neurological manifestations and hemorrhagic phenomena. Convulsion, the main neurological manifestation in these neonates, could be due to encephalitis and intracranial hemorrhage. Cerebral edema may increase permeability of the blood-brain barrier without virus-induced damage of the central nervous system, but a direct virus-induced encephalitis cannot be ruled out. Magnetic resonance imaging showed severe white matter injury that has been well characterized in a 3 stage: cytotoxic brain edema (ischemia), vasogenic edema (reperfusion) and mass reduction (demyelination) [6,16,43].

The intensity of thrombocytopenia was associated with severe neonatal disease as central nervous system hemorrhages, namely scattered cerebrum parenchyma petechiae associated with a clinical context of disseminated intravascular coagulation syndrome.

Cutaneous hyperpigmentation in the convalescence phase was related to maternal CHIKV infection in the first trimester of gestation and usually disappeared around 6–7 months of life [51]. It demonstrates the ability of the virus to cross the barrier of placenta and points out an important sign of infection during early pregnancy.

The main chikungunya laboratory finding reported in the literature, lymphopenia was related to viremia and was defined when the lymphocyte count was lower than 1,000 cells/mm3. However, in this review it was not the most frequent laboratory abnormality; instead, increased levels of alanine aminotransferase and aspartate aminotransferase and hypocalcemia in the neonatal blood were the most often reported. Erythrocyte sedimentation rate was generally increased and correlated with active disease [58]. Thrombocytopenia (platelets <150000 mm3) was a frequent laboratory finding in the cases reported here, associated with the clinical picture of cerebral, gastrointestinal and conjunctival hemorrhages [53,58].

For differential diagnosis of CHIKV vertical transmission with other arboviral diseases sharing similar outcomes during gestation (miscarriage, preterm birth, fetal distress), sepsis should alert neonatologists and evoke chikungunya. Hyperalgesia, difficult to evaluate, and irritability, although common in neonatal CHIKV, were not specific and could be due to neonatal sepsis of other etiology. Although clinical and epidemiological criteria are important for the clinical and therapeutic approach of these neonates, especially in epidemic-affected areas, there are no specific clinical criteria that define neonatal CHIKV infection. Laboratory diagnosis is essential for the confirmation of infection and to differentiate chikungunya from other causes of fever in the neonate.

In only one study a pain scale was used for monitoring the infected neonates [59]. Arthralgia was little reported in the articles evaluated because pain was not assessed, therefore it is important to routinely establish neonatal pain monitoring in neonatal ICUs [58,60,61]. In fact, arthralgia should be assessed in neonates, mainly in those presenting limb edema with engulfing ankles and wrists. It was even proposed that suckling difficulties were due to mandibular painful involvement, mimicking trismus in some cases [6].

In conclusion, this systematic study reports the impact of CHIKV maternal infections during gestation on the fetuses and the newborn infants. The cases included in this review indicate that CHIKV congenital infection can be severe and therefore differential diagnosis of sepsis should be considered in affected areas, regardless of the presence or not of mother’s clinical manifestation. Neonatologists should be aware of late manifestations and babies of symptomatic mothers should be kept in the maternity ward for a week for clinical and laboratory surveillance through serial blood count with platelet count monitoring. Whether the neonates become symptomatic or present with lymphopenia or thrombocytopenia, they should be transferred urgently to a NICU.

This study characterizes the clinical presentation in the neonate of perinatally-acquired CHIKV infection during childbirth, which is critical for a better clinical awareness aimed at diagnosis improvement, early and appropriate therapeutic management. Therapeutic interventions aimed at clearing maternal or early neonatal viremia, such as anti-CHIKV hyperimmune immunoglobulins, should be mandatory to prevent neurological damage and lifelong disability in infected neonates.

Since now we have all the articles, we can contact the authors and prepare a data extraction form with all the variables of interest for a meta-analysis plan of individual data.

## Supporting information

S1 File Prisma checklist. (DOCX)

S2 File Search strategy. (DOCX)

S3 File Quality assessment. (DOCX)

S4 File (PDF)

S5 File (PDF)

S6 File (PDF)

S7 File (PDF)

S8 File (PDF)

S9 File (PDF)

S10 File (PDF)
