# Correction: Vertical transmission of chikungunya virus: A systematic review

**DOI:** 10.1371/journal.pone.0272761

**Published:** 2022-08-03

**Authors:** Fátima Cristiane Pinho de Almeida Di Maio Ferreira, Anamaria Szrajbman Vaz da Silva, Judith Recht, Lusiele Guaraldo, Maria Elisabeth Lopes Moreira, André Machado de Siqueira, Patrick Gerardin, Patrícia Brasil

There is an error in affiliation 5 for author Patrick Gerardin. The correct affiliation 5 is: Centre for Clinical Investigation—Clinical Epidemiology (INSERM CIC1410), CHU Réunion, Saint Pierre, Reunion Island, France.

The following information is missing from the Funding statement: This study was also partially supported by the Coordination for the Improvement of Higher Education Personnel (Coordenação de Aperfeiçoamento de Pessoal de Nível Superior—CAPES) in the form of funds to FCPdADMF [Finance Code 001].
