# Supplementary material for: Vertical transmission of chikungunya virus: A systematic review
Source: PLoS One. 2021 Apr 23;16(4):e0249166. doi: 10.1371/journal.pone.0249166 (PMC8064608; doi:10.1371/journal.pone.0249166)
Supplement: S1 File — (DOCX) [file pone.0249166.s001.docx]

**Appendix 1- search strategy performed in the databases**

| Base | Search |  |
| --- | --- | --- |
| Medline | (((congenital chikungunya) AND (((newborn) OR (((infant OR neonatal))) AND (((mother-to-child OR congenital OR vertical transmission)))) AND chikungunya) | 44 |
| Lilacs | mh:(chikungunya) AND mh:(newborn)  (mj: (“chikungunya fever”) AND (“newborn”)) AND limit: (“humans”) | 6 |
| Web of science | (“chikungunya virus”) AND (“mother to child transmission”)  (“congenital chikungunya”) OR (“neonatal chikungunya”)  (“Chikungunya”) AND (“newborn”)  (“Chikungunya”) AND (“pregnant”)  (“Chikungunya”) AND (“vertical transmission”) | 26 |
| Scopus | “Neonatal chikungunya” or “congenital chikungunya”  "chikungunya" AND "vertical transmission" AND "perinatal infection"  chikungunya virus and neonatal chikungunya | 35 |
| Google Scholar | Chikungunya AND newborn  Chikungunya AND pregnancy | 29 |
