# Supplementary material for: Vertical transmission of chikungunya virus: A systematic review
Source: PLoS One. 2021 Apr 23;16(4):e0249166. doi: 10.1371/journal.pone.0249166 (PMC8064608; doi:10.1371/journal.pone.0249166)
Supplement: S6 File — (PDF) [file pone.0249166.s006.pdf]

Lorem Ipsum

Lorem ipsum dolor sit amet, consectetur adipiscing elit. Mauris maximus fringilla ligula, in malesuada erat tempor ac. Quisque dapibus posuere turpis, vel aliquam massa vehicula non.

Quality assessment of the studies included in the systematic review based on MINORS (Slim et al., 2003)

**Articles**	M1	M2	M3	M4	M5	M6	M7	M8	M9	M10	M11	M12
Senanayake	0	2	2	2	0	2	1	0	na	na	na	na
Lenglet	2	2	2	2	0	2	2	0	na	na	na	na
Paquet	2	0	2	2	0	2	2	2	na	na	na	na
Torres	2	2	2	1	0	2	2	2	na	na	na	na
Fritel	2	2	2	2	0	2	2	2	2	2	1	2
Ramful	2	2	2	2	0	2	0	2	na	na	na	na
Villamil-Gomez	2	2	2	2	0	2	2	0	na	na	na	na
Pinzon-Redondo	2	2	2	2	0	2	0	2	na	na	na	na
Maria	0	0	2	2	0	0	2	0	na	na	na	na
Gerardin_2008	2	2	2	2	0	2	0	2	2	2	0	1
Gerardi_2014	2	2	2	2	2	2	1	2	2	2	2	2
Dorleans, 2018	2	2	2	2	1	2	2	2	na	na	na	na
Ramful, 2007	0	0	2	2	0	2	2	0	na	na	na	na
Mangalgi,2011	0	0	0	2	0	2	2	0	na	na	na	na

*The items are scored: 0 (not reported), 1 (reported but inadequate) or 2 (reported and adequate); na = not aplicable(non-comparative studies)

Methodological items for non-randomized studies

M1 . A clearly stated aim:
the question addressed should be precise and relevant in the light of available literature

M2. Inclusion of consecutive patients:
all patients potentially fit for inclusion (satisfying the criteria for inclusion) have been included in the study during the study period (no exclusion or details about the reasons for exclusion)

M3. Prospective collection of data:
data were collected according to a protocol established before the beginning of the study

M4.Endpoints appropriate to the aim of the study:
unambiguous explanation of the criteria used to evaluate the main outcome which should be in accordance with the question addressed by the study.
Also, the endpoints should be assessed on na intention-to-treat basis.

M5.Unbiased assessment of the study endpoint:
blind evaluation of objective endpoints and double-blind evaluation of subjective endpoints. Otherwise the reasons for not blinding should be stated

M6.Follow-up period appropriate to the aim of the study:
the follow-up should be sufficiently long to allow the assessment of the main endpoint and possible adverse events

M7.Loss to follow up less than 5%:
all patients should be included in the follow up. Otherwise, the proportion lost to follow up should not exceed the proportion experiencing the major endpoint

M8.Prospective calculation of the study size:
information of the size of detectable difference of interest with a calculation of 95% confidence interval, according to the expected incidence of the outcome event, and information about the level for statistical significance and estimates of power when comparing the outcomes

Additional criteria in the case of comparative study

M9.An adequate control group:
having a gold standard diagnostic test or therapeutic intervention recognized as the optimal intervention according to the available published data

M10.Contemporary groups:
control and studied group should be managed during the same time period (no historical comparison)

M11.Baseline equivalence of groups:
the groups should be similar regarding the criteria other than the studied endpoints. Absence of confounding factors that could bias the interpretation of the results

M12.Adequate statistical analyses:
whether the statistics were in accordance with the type of study with calculation of confidence intervals or relative risk

Fig1.tif This is a preview of your figure rendered on a simulated PLOS journal page.

Maecenas ac est sit amet odio sollicitudin euismod. In risus odio, convallis a neque ac, varius ultricies arcu. Vestibulum et quam iaculis, ultricies odio et, molestie magna. Suspendisse vehicula purus id turpis eleifend, et convallis dui dignissim. Praesent tempus elit a metus sollicitudin, sed fringilla nulla porttitor. Nullam in tempus massa. Nunc maximus magna massa, nec volutpat risus rhoncus ut. Fusce quis ante sem. Aenean nulla nibh, tempus sit amet rhoncus at, eleifend vel risus. Sed dictum, sem ultrices elementum pharetra, lacus diam volutpat orci, scelerisque semper dui lacus ut enim.

Suspendisse in nunc id lacus commodo consequat. Proin semper aliquam varius. Fusce vitae neque aliquam nisi ultrices sodales vitae ut enim. Vivamus nec dictum ipsum. Sed condimentum ante eu urna tincidunt tincidunt. In ac lacus nec ipsum viverra volutpat posuere vel lacus. Class aptent taciti sociosqu ad litora torquent per conubia nostra, per inceptos himenaeos. Morbi rhoncus ipsum quis lorem hendrerit, at vulputate massa tempus. Ut arcu nisl, gravida vitae risus ultricies, porta venenatis massa. Cras dignissim, enim at faucibus aliquam, sapien nisl eleifend dolor, vel mollis nulla nisi id ipsum. Pellentesque vehicula ultricies risus sit amet faucibus. Praesent sit amet mi ac est faucibus accumsan. Praesent pulvinar sit amet orci auctor feugiat.

Phasellus vitae congue est. Duis rutrum iaculis nunc, sed sollicitudin neque eleifend nec. Pellentesque ac nisi eget tortor imperdiet sagittis ut in orci. Mauris porta convallis euismod. Donec in ultricies urna, nec interdum lectus. Nullam sit amet finibus augue, eget rutrum metus. Nam faucibus, urna ac finibus eleifend, neque nisi lobortis ante, at pharetra purus purus sed urna. Curabitur sit amet dui at enim porta posuere non vehicula ligula. Suspendisse potenti. Vestibulum arcu magna, vulputate a massa ac, molestie tincidunt dui.

Donec id tempus lacus, sed tristique nulla. Nullam rutrum risus ut pharetra porttitor. Nam mattis dolor erat, sed volutpat est mattis sed. Suspendisse eu porta tellus. Cras gravida velit sed maximus fermentum. Fusce vitae metus commodo, sagittis nunc sed, faucibus nunc. Integer iaculis quam mattis, luctus neque in, viverra magna.

Nulla rhoncus feugiat orci, quis posuere ligula ornare at. Integer vel sagittis risus. Donec semper metus nec finibus accumsan. Mauris sit amet suscipit ante. Aliquam accumsan, nisl vitae vulputate elementum, turpis nibh varius urna, vel bibendum nulla nunc ac quam. Aenean malesuada egestas maximus. Pellentesque faucibus, odio at tincidunt ullamcorper, eros nisi pellentesque mi, non blandit sapien neque quis lectus.
