# Supplementary material for: Vertical transmission of chikungunya virus: A systematic review
Source: PLoS One. 2021 Apr 23;16(4):e0249166. doi: 10.1371/journal.pone.0249166 (PMC8064608; doi:10.1371/journal.pone.0249166)
Supplement: Supplementary file 10 [file pone.0249166.s003.docx]

Quality assessment of the studies included in the systematic review based on MINORS (Slim et al., 2003)

Articles	M1	M2	M3	M4	M5	M6	M7	M8	M9	M10	M11	M12
Senanayake	0	2	2	2	0	2	1	0	na	na	na	na
Lenglet	2	2	2	2	0	2	2	0	na	na	na	na
Paquet	2	0	2	2	0	2	2	2	na	na	na	na
Torres	2	2	2	1	0	2	2	2	na	na	na	na
Fritel	2	2	2	2	0	~~2~~	~~2~~	2	2	2	1	2
Ramful	2	2	2	2	0	2	0	2	na	na	na	na
Villamil-Gomez	2	2	2	2	0	2	2	0	na	na	na	na
Pinzon-Redondo	2	2	2	2	0	2	0	2	na	na	na	na
Maria	0	0	2	2	0	0	2	0	na	na	na	na
Gerardin_2008	2	2	2	2	0	2	0	2	2	2	0	1
Gerardi_2014	2	2	2	2	2	2	1	2	2	2	2	2
Dorleans, 2018	2	2	2	2	1	2	2	2	na	na	na	na
Ramful, 2007	0	0	2	2	0	2	2	0	na	na	na	na
Mangalgi,2011	0	0	0	2	0	2	2	0	na	na	na	na

* The items are scored: 0 (not reported), 1 (reported but inadequate) or 2 (reported and adequate); na =not aplicable(non-comparative studies)

Methodological items for non-randomized studies

M1 . A clearly stated aim:
the question addressed should be precise and relevant in the light of available literature

M2. Inclusion of consecutive patients:
all patients potentially fit for inclusion (satisfying the criteria for inclusion) have been included in the study during the study period (no exclusion or details about the reasons for exclusion)

M3. Prospective collection of data:
data were collected according to a protocol established before the beginning of the study

M4.Endpoints appropriate to the aim of the study:
unambiguous explanation of the criteria used to evaluate the main outcome which should be in accordance with the question addressed by the study.
Also, the endpoints should be assessed on na intention-to-treat basis.

M5.Unbiased assessment of the study endpoint:
blind evaluation of objective endpoints and double-blind evaluation of subjective endpoints. Otherwise the reasons for not blinding should be stated

M6.Follow-up period appropriate to the aim of the study:
the follow-up should be sufficiently long to allow the assessment of the main endpoint and possible adverse events

M7.Loss to follow up less than 5%:
all patients should be included in the follow up. Otherwise, the proportion lost to follow up should not exceed the proportion experiencing the major endpoint

M8.Prospective calculation of the study size:
information of the size of detectable difference of interest with a calculation of 95% confidence interval, according to the expected incidence of the outcome event,
and information about the level for statistical significance and estimates of power when comparing the outcomes

Additional criteria in the case of comparative study

M9.An adequate control group:
having a gold standard diagnostic test or therapeutic intervention recognized as the optimal intervention according to the available published data

M10.Contemporary groups:
control and studied group should be managed during the same time period (no historical comparison)

M11.Baseline equivalence of groups:
the groups should be similar regarding the criteria other than the studied endpoints. Absence of confounding factors that could bias the interpretation of the results

M12.Adequate statistical analyses:
whether the statistics were in accordance with the type of study with calculation of confidence intervals or relative risk
